# Transdermal Estrogen Therapy Improves Gains in Skeletal Muscle Mass After 12 Weeks of Resistance Training in Early Postmenopausal Women

**DOI:** 10.3389/fphys.2020.596130

**Published:** 2021-01-18

**Authors:** Tine Vrist Dam, Line Barner Dalgaard, Steffen Ringgaard, Frank Ted Johansen, Mads Bisgaard Bengtsen, Maike Mose, Katrine Meyer Lauritsen, Niels Ørtenblad, Claus H. Gravholt, Mette Hansen

**Affiliations:** ^1^Department of Public Health, Aarhus University, Aarhus, Denmark; ^2^The MR Research Centre, Aarhus University Hospital, Aarhus, Denmark; ^3^Department of Endocrinology and Internal Medicine, Aarhus University Hospital, Aarhus, Denmark; ^4^Department of Sports Science and Clinical Biomechanics, University of Southern Denmark, Odense, Denmark; ^5^Department of Molecular Medicine, Aarhus University Hospital, Aarhus, Denmark

**Keywords:** exercise, estradiol, menopause, hormone replacement therapy, muscle hypertrophy, muscle strength, strength training

## Abstract

**Context:**

Women show an accelerated loss of muscle mass around menopause, possibly related to the decline in estrogen. Furthermore, the anabolic response to resistance exercise seems to be hampered in postmenopausal women.

**Objective:**

We aimed to test the hypothesis that transdermal estrogen therapy (ET) amplifies the skeletal muscle response to resistance training in early postmenopausal women.

**Design:**

A double-blinded randomized controlled study.

**Setting:**

Department of Public Health, Aarhus University, Denmark.

**Participants:**

Thirty-one healthy, untrained postmenopausal women no more than 5 years past menopause.

**Intervention(s):**

Supervised resistance training with placebo (PLC, *n* = 16) or transdermal ET (*n* = 15) for 12 weeks.

**Main Outcome Measure(s):**

The primary outcome parameter was a cross-sectional area of quadriceps femoris measured by magnetic resonance imaging, and secondary parameters were fat-free mass (dual-energy X-ray absorptiometry), muscle strength, and functional tests.

**Results:**

The increase in muscle cross-sectional area was significantly greater in the ET group (7.9%) compared with the PLC group (3.9%) (*p* < 0.05). Similarly, the increase in whole-body fat-free mass was greater in the ET group (5.5%) than in the PLC group (2.9%) (*p* < 0.05). Handgrip strength increased in ET (*p* < 0.05) but did not change in the PLC group. Muscle strength parameters, jumping height, and finger strength were all improved after the training period with no difference between groups.

**Conclusion:**

The use of transdermal ET enhanced the increase in muscle mass in response to 12 weeks of progressive resistance training in early postmenopausal women.

## Introduction

Menopause is defined by the permanent cessation of menstruation at an average age of 51 years. The transition into menopause is characterized by a significant decline in circulating estrogens (Vitale et al., 2016). The literature shows an accelerated loss of muscle mass and strength in women around the time of menopause, which is not observed in age-matched men (Phillips et al., 1993). Furthermore, compared with age-matched men, postmenopausal women show reduced anabolic stimuli to resistance training (Bamman et al., 2003), and the muscle protein synthesis rate in response to feeding is also reduced in postmenopausal women (Smith et al., 2008). It is suggested that the observed sex differences are related to the sex hormonal changes women experience around menopause (Phillips et al., 1993; Bamman et al., 2003; Baltgalvis et al., 2010; Lowe et al., 2010; Hansen et al., 2012). In light of, women nowadays are expected to live more than 1/3 of their life in menopause; more knowledge in this field is wanted to combat some of the degenerative changes experienced during and after the transition into menopause.

Endogenous estrogen mediates its action by binding to the estrogen receptors (ERs): ER-α, ER-β, or the membrane-bound G protein-coupled estrogen receptor 1 (Baltgalvis et al., 2010). By estrogen binding to ER-α or ER-β, the receptors are activated and translocate to the nucleus, where they function as transcription factors to control gene transcription (Baltgalvis et al., 2010). In addition to the genomic effects, estrogen also signals through rapid non-genomic pathways by binding to G protein-coupled estrogen receptor 1, initiating intracellular signaling cascades with, e.g., antioxidant effects (Mooradian, 1993). Estrogen receptors have been localized within skeletal muscle (Wiik et al., 2009). This suggests a direct role of estrogens in skeletal muscle tissue. In support, data indicate that estrogen reduces muscle damage and improves satellite cell activation and proliferation, as well as myosin function (Tiidus, 2005; Enns et al., 2008). Nevertheless, animal and muscle cell studies show divergent results when it comes to estrogen effects on skeletal muscle mass, muscle function, and muscle protein synthesis and breakdown (Toth et al., 2001; Pampusch et al., 2008; Greising et al., 2009; Hansen and Kjaer, 2014). The reported differences are likely explained by differences between species and related to differences in sex hormone profiles and emphasizes the need for human studies to get a better understanding of estrogen’s influence on human skeletal muscle mass and strength and the adaptive response to resistance training in postmenopausal women.

Resistance training is reported to effectively counteract the loss of muscle mass during aging (Raguso et al., 2006), but postmenopausal women may be less sensitive to anabolic stimuli (e.g., resistance training) due to their low estrogen level (Bamman et al., 2003; Hansen et al., 2012; Hansen, 2018). Randomized controlled studies investigating adaptations to resistance training in postmenopausal women using estrogen replacement therapy (ET) are lacking. A few older human studies have investigated the effect of hormone replacement therapy (HT) on skeletal muscle mass in postmenopausal women with mixed results and at high risk of bias (Sipila et al., 2001; Taaffe et al., 2005; Finni et al., 2011; Qaisar et al., 2013; Javed et al., 2019). HT contains both estrogen and synthetic progesterone (gestagen), and the effect of HT may be different from the effect of estrogen or gestagen alone. The administration method, dose and type of HT, age of the participants, and different types of training interventions make comparisons between studies difficult. For instance, oral administration of 17-β estradiol leads to reductions in insulin-like growth factor-1 (IGF-1) and an increase in growth hormone, which is not reported when using transdermal estrogen (dos Reis et al., 2003). In addition, HT administration seems to have more positive effects on the muscle protein balance when the use of HT is initiated soon after the transition into menopause compared with delayed (Park et al., 2019). Taken together, our current knowledge about the isolated role of estrogen on the adaptive response to resistance training in postmenopausal women is sparse. Therefore, we aimed at investigating if the use of transdermal estrogen therapy influences the response in muscle mass to 12 weeks of progressive resistance training in postmenopausal women. We hypothesized that the use of transdermal estrogen therapy would lead to greater gains in muscle mass and strength in response to resistance training compared with placebo. The primary outcome parameter is the cross-sectional area of quadriceps femoris, measured by magnetic resonance imaging (MRI), and secondary parameters are fat-free mass, muscle strength, and functional tests.

## Materials and Methods

### Experimental Design

**FIGURE 1:**
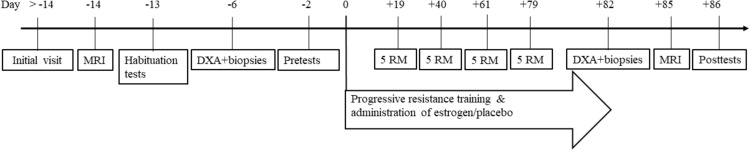
Overview of protocol. Initial visit included measured height, weight, questionnaire, and a screening blood sample. Habituation, pre- and post-test included: five RM (leg press, knee extension, and knee flexion), maximum voluntary isometric contraction, electromyogram, functional tests (sit-and-raise, 6 min walk, countermovement jump, hand and finger strength, and nine-hole-peg-test). MRI, magnetic resonance imaging of legs and DXA, whole body dual-energy-X-ray absorptiometry scan.

This study was conducted as a double-blinded randomized controlled study, where early postmenopausal women were randomly allocated to receive either placebo or transdermal estrogen therapy for 12 weeks. During the intervention period, both groups performed supervised resistance training three times per week (36 sessions). An overview of the study is shown in Figure 1. Before the intervention, subjects who completed an MRI scan of the thigh muscles were familiarized with the training exercises, strength and muscle function tests, and physical activity level, and dietary food intake was registered. Lastly, a dual-energy X-ray absorptiometry (DXA) scan and a resting muscle biopsy were obtained after an overnight fast, and baseline measures of muscle strength and functional tested were collected. On the last day of the intervention period (day + 82), the participants arrived at the laboratory after an overnight fast for a DXA scan and a muscle biopsy before performing the last training session. Four days after the last training session (day + 86), all the tests performed before the intervention were repeated (Figure 1).

### Participants

Healthy, untrained early postmenopausal women were recruited through local newspapers. As part of the screening process, the subjects received information about the study and completed a questionnaire regarding inclusion/exclusion criteria and physical activity level. Furthermore, blood pressure, weight, and height were measured, and blood samples were collected for analysis of sex hormones and health-related parameters. The inclusion criteria were as follows: (1) no menstruation for at least 6 months, (2) last menstruation less than 5 years ago, and (3) minimum 40 years of age. Exclusion criteria were (1) more than 2 h of moderate- to high-intensity training per week in the last year, (2) systematic resistance training (more than five times in the last year), (3) use of hormone therapy within the last 6 months, (4) muscle, joint, or metabolic diseases, which might influence the result of the study, (5) high blood pressure, (6) smoking, and (7) present or previous cancer diagnosis.

**FIGURE 2:**
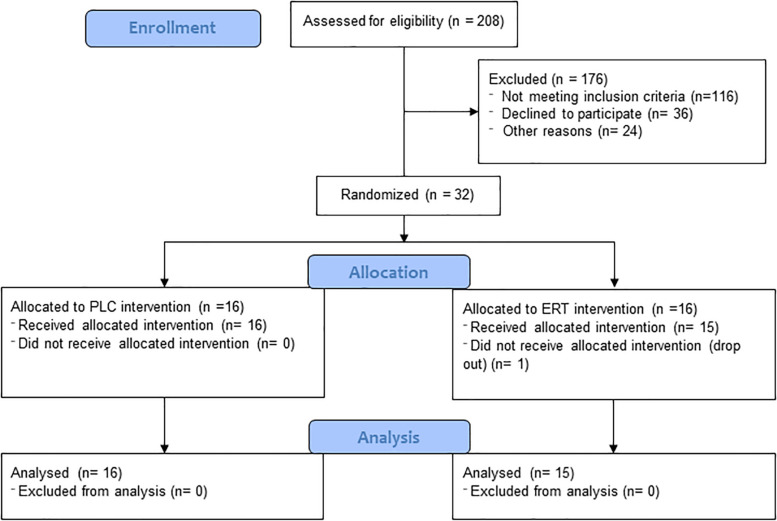
Flow diagram reflecting the recruitment and allocation process.

Thirty-two women were randomly allocated to an intervention group by the principal investigator based on a recruitment order. The participants and personal test were blinded to the allocation. One participant did not show up to the initial test and start of the intervention, but the remaining 31 postmenopausal women (PLC *n* = 16, ET *n* = 15) completed the intervention period and tests. Subject characteristics are shown in Table 1. The two groups did not differ in the following characteristics at baseline: age, weight, height, BMI, time since last menstruation, and body fat mass (FM). A flow diagram reflecting the recruitment and allocation process is shown in Figure 2.

**TABLE 1:** Subject characteristics at baseline.

**Measurement**	**Estrogen (*n* = 15)**		**Placebo (*n* = 16)**		***P*-value**
Age (years)	53.7	± 4.1	54.6	± 3.8	0.56
Weight (kg)	70.2	± 10.9	69.2	± 9.6	0.79
Height (cm)	169	± 6	168	± 8	0.63
BMI (kg/m^2^)	24.6	± 3.5	24.6	± 2.9	0.99
Time since last menstruation (months)	34.1	± 18.3	35.3	± 20.1	0.86
Body fat (kg)	26.4	± 8.0	25.5	± 6.1	0.74

*Values are presented as mean ± SD.*

The study was carried out in accordance with the Helsinki Declaration, approved by the local ethical committee of Region Midtjylland (j. no. 1-10-72-234-16) and registered at clinical.trials.gov (ID: nct03020953). The study was reported to the Danish Data Protection Agency. All subjects provided written consent before the intervention.

### Hormone Administration

Transdermal 17-β estradiol or placebo patches were distributed to the subjects in a closed non-transparent envelope, and subjects were instructed to place a patch on the skin on the lower part of the abdominal region at the start of the intervention. The subjects were instructed to renew the patch twice a week. The ET patches were releasing 100 μg 17-β-estradiol per 24 h (Vivelle dot, Novartis Healthcare, Copenhagen, Denmark). The placebo patches (Matas blister patch, size small, Allerød, Denmark) and the ET patches both had a neutral, unlabeled appearance. Participants in the ET group were offered 10 days of treatment with progesterone (Provera, Pfizer, United States, 10 mg per day) after the intervention.

### Training Protocol

The training program was constructed after the guidelines provided by the American College of Sports Medicine (American College of Sports Medicine, 2009). Each training session was initiated by a standardized warm-up consisting of 5 min of light cycling, light running, or light rowing, followed by six resistance exercises. The primary focus in the training intervention was on the lower extremities and included three leg exercises in resistance training machines: leg press (Johnson, United States), knee extension (Nordic Gym, United Kingdom), and knee flexion (Nordic Gym, United Kingdom). The three other exercises were dumbbell triceps extension, sit-ups, and back extensions. The program was built progressively to avoid overload injuries, as follows: week 1: 2 sets/15 repetitions at 15 repetition maximum (RM); week 2: 2 sets/12 repetitions at 12 RM; week 3–6: 3 sets/12 repetitions at 12 RM; week 7–10: 3 sets/10 repetitions at 10 RM; week 11–12: 4 sets/8 repetitions at 8 RM. All training sessions were supervised to ensure proper load resistance during the exercises, technique and adherence to the program. Besides the supervised training, the subjects were instructed to maintain their normal physical activity level and not engage in any other types of strenuous exercise.

### Magnetic Resonance Images

Changes in muscle cross-sectional area (mCSA) were evaluated by MRI scans performed before and after the training intervention. All scans were done by a 3-T whole-body magnetic resonance scanner (Magnetom Skyra; Siemens, Erlangen, Germany) at the magnetic resonance center at Aarhus University Hospital, Aarhus, Denmark. Physical habituation tests and biopsies were at least 48 h before the MRI scan to minimize the risk of water retention in the muscle (Kristiansen et al., 2014). Subjects were placed in a supine position. The images included both legs and were collected using two 18-element anterior receive coils covering the thighs. After an initial frontal scout scan, a transversal fast spin-echo Dixon scanning was performed with the following parameters used: number of slices = 60, slice thickness = 3 mm, slice gap = 7 mm, field of view = 460 × 273 mm, acquisition matrix = 512 × 243, repetition time = 979 ms, echo time = 11 ms, echo train length = 5, pixel bandwidth = 305 Hz/pixel, and scan time = 3:42 min. Calculations of whole-muscle CSA were performed by manually segmenting the muscles at two axial positions obtained at 15 and 25 cm above the tibia plateau. All images were processed three times by the same researcher in a blinded fashion using the home-written program “Siswin.”

### Dual Energy X-ray Absorptiometry and Anthropometrics

A DXA scan was performed to determine the change in body composition (GE Lunar DXA scan, GE Healthcare, Madison, WI, United States), and the system’s software package (enCORE software v16.0, GE Healthcare, Madison, WI, United States) was used to determine FM, fat-free mass (FFM), bone mass, bone mass density, visceral FM, body fat %, and android/gynoid fat ration (A/G ratio).

### Maximal Isometric Strength and Surface Electromyography Measures and Analysis

Subjects were seated in an isokinetic dynamometer (Humac Norm, CSMi, Stoughton, Massachusetts, United States) with a hip angle of 90°. Before the pretesting, a familiarization session was completed to lower the learning effect. The subjects performed 3–5 maximal isometric voluntary contractions (MVIC) for the knee extensors and flexors at a knee angle of 70° and 20°, respectively. The subjects received visual feedback and standardized verbal encouragement during each attempt. All contractions were separated by > 30 s of rest. The right leg was tested, and the attempt having the highest peak torque was used for further analysis. Concomitantly, surface electromyography (EMG) electrodes (Ambu Blue Sensor N, AMBU, Ballerup, Denmark) were attached to musculi (mm.) vastus lateralis and biceps femoris. After shaving and cleaning the skin, electrodes were placed 2 cm apart and in relation to anatomical marks.

### Analysis

All data were sampled using TeleMyo Direct Transmission System and MyoResearch Software (Noraxon, Scottsdale, Arizona, United States) at 1,500 Hz, and analyses were performed using custom-made software. Torque data were filtered using a Butterworth low-pass filter (cutoff frequency: 6 Hz) and gravity corrected. Contraction onset was defined when torque increased above 7.5 Nm15, and MVIC was defined as the peak torque measurement. Maximal rate of force development (RFDmax) was defined as the steepest slope between onset and MVIC. Additionally, RFD was determined as the average slope from 0 to 100 ms and from 0 to 200 ms after the onset of contraction. EMG signals were full-wave rectified and similarly low-pass filtered.

### Five Repetition Maximum Strength Tests

The five RM tests were carried out in connection with training sessions 9, 18, 27, and 35 by substitution of the leg exercises with the five RM tests and at the posttest after the intervention. After 5 min of warm-up, participants were instructed to make 10 repetitions with a moderate weight. Each participant’s five RM was predicted using the Brzyckis equation (Brzycki, 1993). If the subject successfully performed more than five repetitions, the load was increased until the subject could no longer perform more than five accepted repetitions. In general, five RM was reached within three trials. Three minutes of rest was given between sets, and standardized verbal encouragement was provided.

### Functional Tests

The countermovement jump (CMJ) test was performed on a speed force-platform (Swift performance, Wacol, Australia). Participants were instructed to perform a maximal vertical CMJ with their hands placed on their hips. Each participant was allowed three attempts. The highest jump was selected for further analysis. Handgrip strength and finger strength (kilogram) of the right hand were assessed using a North Coast Medical dynamometer (California, United States). Three attempts for each test was given with a rest between every trial. To address a possible effect on endurance and walking speed, the 6 min walk test was applied. The subjects were asked to walk as fast as possible along a marked indoor corridor, and the distance during 6 min was recorded (Bellet et al., 2012). Lastly, a 30 s sit-to-stand test, where the maximal number of times each subject could rise up from a chair and sit down again, arms over crossed, within 30 s was recorded (Jones et al., 1999). To measure dexterity, the nine-hole peg test was applied (Kellor et al., 1971). Each subject had two attempts.

### Four-Day Dietary Record

Before and during the last week of the training intervention, a 4 days dietary record was completed by all participants on 3 consecutive week-days and 1 weekend-day. Subjects were instructed to eat habitually and register it in the software program MADLOG (MADLOG aps, Kolding, Denmark). The data were analyzed for total daily energy consumption, protein, fat, and carbohydrate intake. Three subjects (ET = 1, PLC = 2) were excluded from the nutrition analysis due to incomplete reporting, and 17 (ET = 9, PLC = 8) were further excluded because they did not meet the criteria of Goldberg cutoff limits (energy intake/basal metabolic rate, < 1.06) (Goldberg et al., 1991).

### Physical Activity Level

Before and in the last week of the intervention, each participant had an accelerometer (GT3X Actigraph accelerometers, Pensacola, Florida, United States) positioned on the right hip at the anterior axillary line for 7 consecutive days, keeping the accelerometer on at all time. Data were collected in three axes; vertical, horizontal right-left, and horizontal front-back. In addition, the output included the vector magnitude, which was based on the three individual axes. The raw acceleration data were collected at 80 Hz. Data were downloaded to Actilife (version 6), using 60 s epochs, and exported to a custom-made software program, “Actianalyzer” (Cuno Rasmussen, Department of Public Health, Aarhus University).

### Blood Samples

As part of the screening procedure, blood samples were obtained from the antecubital vein after overnight fasting and analyzed for standard blood parameters, including sex hormones. Alanine transaminase, aspartate aminotransferase, hemoglobin, creatine kinase, calcium, sodium, potassium, thrombocytes, thyroid-stimulating hormone, leukocytes, C-reactive protein, albumin, insulin, and 25-hydroxy vitamin D were all within the normal range. Furthermore, estradiol, testosterone, sex hormone-binding globulin (SHBG), progesterone, follicle-stimulating hormone (FSH), luteinizing hormone (LH), IGF-1, and IGF-1-BP3 were determined before and after the intervention. The samples were centrifuged and stored for later analysis.

Samples were analyzed by standard procedures at the Department of Clinical Biochemistry, the Department of Medical Endocrinology at Aarhus University Hospital, Aarhus, Denmark, or the Department for Public Health, Aarhus University, Denmark. Estradiol levels below the detection level were set to the detection level (15 nmol/L) in statistical analyses.

### Muscle Biopsy and Myosin Heavy Chain Analysis

Muscle biopsies were obtained under local anesthesia after overnight fasting before and after the intervention from m. vastus lateralis approximately 1/3 above patella plateau to the ilia crest using the Bergström needle technique. The post-intervention biopsy was taken 5 cm above the incision hole from the baseline biopsy. A part of the biopsy was immediately frozen in liquid nitrogen and stored at –80° until analysis. Myosin heavy chain (MHC) composition was determined from homogenate using gel electrophoresis as previously described (Betto et al., 1986) and modified for humans (Ortenblad et al., 2000).

### Statistical Analysis

The sample size was determined by assuming ET to induce a 20% greater increase in muscle growth related to ET which has been shown to increase myofibrillar protein synthesis rate in response to resistance exercise by 30% (Hansen et al., 2012). By including 14 subjects in each group, it should be possible to detect the difference between groups with a power of 80%, a significance level of 0.05, and a variation estimated to be 8%.

The statistical analysis was carried out using a linear mixed model (STATAIC 15, StataCorp, College Station, TX). Data were tested for normal distribution of residuals by visual inspection and examination of QQ plots, and if absent, the appropriate transformation was carried out, including log and square root transformation. All repeated measurements, except serum hormone levels, were analyzed with time, pre and post, and group, ET and PLC as the fixed effects. As a random effect variable, the subject ID was added. Sex hormonal parameters were not normally distributed, and Mann–Whitney–Wilcoxon tests were performed to compare pre- to post-values. To test for significant changes in sex hormones from pre- to post-intervention within each group, a Wilcoxon signed-rank test was performed.

All data are presented as mean ± *SD*. The statistically significant level was set at *p* < 0.05. GraphPad Prism 8 (GraphPad Software, San Diego) was used as graphing software.

## Results

All 31 participants completed the 36 supervised training sessions as planned (100% adherence). Three women experienced menstrual bleeding halfway through the intervention. Two of them received ET, whereas one was in the PLC group.

### Changes in Muscle Mass—Fat-Free Mass and Muscle Cross-Sectional Area

**FIGURE 3:**
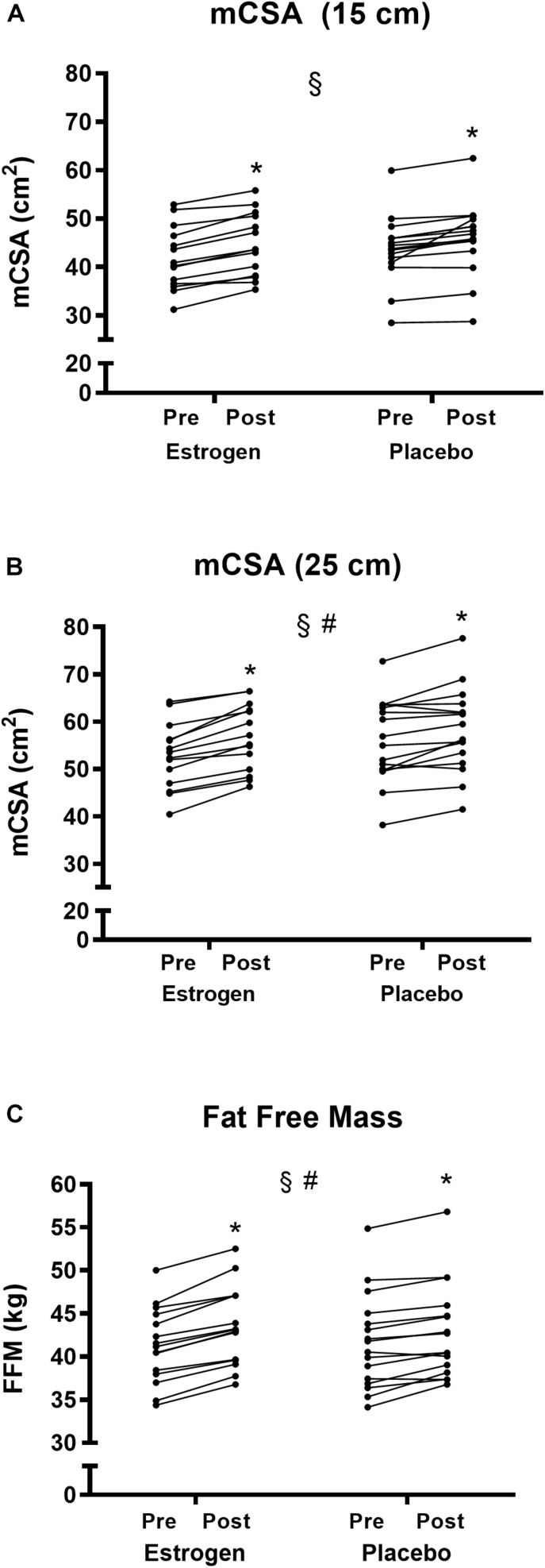
**(A)** Muscle cross-sectional area determined 15 cm above patella plateau (mCSA 15 cm) **(B)** mCSA determined 25 cm measured by magnetic resonance imaging and **(C)** Whole-body fat-free mass measured by dual-energy-x-ray absorptiometry before and after 12 weeks of resistance training in the individual participants in the ET and PLC groups, respectively. ^#^Group × time interaction (*p* < 0.05). ^§^Training effect (*p* < 0.05). *Different from pre- in the individual group (*p* < 0.05).

After the training period, the DXA revealed a significant increase in whole-body FFM in both the ET group (41.3 ± 4.4 to 43.5 ± 4.6 kg) and the PLC group (41.6 ± 5.5 to 42.9 ± 5.4 kg) (*p* < 0.05). The increase in FFM was greater in the ET group than in the PLC group (5.5 vs. 2.9%, *p* < 0.05) (Figure 3A).

The mCSA of quadriceps femoris increased significantly for both regions (15 and 25 cm above the tibia plateau) (*p* < 0.05). There was a significantly greater increase in mCSA (25 cm) in the ET group compared with that in the PLC group (*p* < 0.05) (Figure 3C). In the PLC group, mCSA (25 cm) increased by 3.9% (56.0 ± 8.8 to 58.2 ± 8.9 mm^2^) (*p* < 0.05), whereas the increase in the ET group was 7.4% (52.8 ± 7.0 to 56.7 ± 7.0 mm^2^) (*p* < 0.05). No significant group difference was detected at mCSA (15 cm) (*p* = 0.23) (Figure 3B). mCSA (15 cm) in the PLC group increased by 4.8% (43.7 ± 6.9 to 45.8 ± 7.4 mm^2^) (*p* < 0.05), whereas the increase was 6.7% in the ET group (41.8 ± 6.5 to 44.6 ± 6.5 mm^2^) (*p* < 0.05).

### Changes in Myosin Heavy Chain Distribution

At baseline, there was a tendency toward a greater proportion of MHC I in the PLC group compared with the ET group (*p* = 0.07). The relative distribution of MHC proteins was not significantly changed after the training period, and no significant interaction effect was observed for any of the MHC fractions. However, there was a numeric downregulation of MHC IIx in both groups, but the change was only significant in the PLC group (*p* < 0.05) (Table 2).

**TABLE 2:** Myosin heavy chain distribution.

	**Estrogen (*n* = 14)**					**Placebo (*n* = 14)**						
	**Pre**		**Post**		***P*-value**	**Pre**		**Post**		***P*-value**	**Training**	**Interaction**
MHC 1 (%)	47.8	± 12.1	54.4	± 10.6	0.09	58.4	± 18.3	60.1	± 15.2	0.69	0.11	0.39
MHC IIa (%)	46.3	± 11.8	42.6	± 9.3	0.35	38.3	± 17.1	38.6	± 14.9	0.95	0.39	0.51
MHC IIX (%)	5.9	± 8.3	3.0	± 2.8	0.57	3.3	± 4.1	1.3	± 2.2	**0.05**	0.52	0.48

*Values are presented as mean ± SD. The p-value at 0.05 is reported in bold.*

### Circulating Sex Hormones

At baseline, no differences between groups were observed in FSH, LH, testosterone, and SHBG (*p* > 0.05) (Table 3). At baseline, three participants from the ET group and nine participants from the PLC group had estrogen levels lower than the detection level (< 15 nmol/L). The intervention increased the level of estradiol in the ET group, whereas no significant change was observed in the PLC group. Furthermore, after the intervention, seven women in the PLC group had estradiol levels below the detection level in contrast with the ET group, where the estradiol level was enhanced markedly above the detectable level. The level of LH was significantly lowered after the intervention in the ET group (*p* < 0.01) but not in the PLC group (*p* = 0.45, interaction *p* < 0.01). FSH decreased significantly more in response to the intervention in the ET group compared with the PLC group (*p* < 0.01). Testosterone and SHBG did not change in response to the intervention, and no training × treatment interaction was observed.

**TABLE 3:** Level of sex hormones.

	**Estrogen (*n* = 15)**					**Placebo (*n* = 16)**						
**Hormone**	**Pre**		**Post**		***P*-values**	**Pre**		**Post**		***P*-values**	**Training**	**Interaction**
FSH (IU/L)	90.9	± 26.2	53.1	± 21.3	**<0.001**	93.5	± 32.3	86.0	± 32.7	**<0.01**	**<0.01**	**<0.001**
LH (IU/L)	44.1	± 9.2	31.8	± 12.0	**<0.01**	39.6	± 10.3	39.2	± 11.1	0.45	**<0.01**	**<0.01**
Estradiol (nmol/L)	69.5	± 100.6	318.0	± 228.1	**<0.001**	19.2	± 6.3	25.5	± 30.6	0.41	**<0.001**	**<0.001**
Testosterone (nmol/L)	1.0	± 0.3	0.9	± 0.4	0.82	0.8	± 0.3	0.8	± 0.3	0.98	0.78	0.77
SHBG (nmol/L)	79.4	± 24.2	84.4	± 24.3	0.71	81.7	± 36.0	84.2	± 35.1	0.53	0.98	0.82

*Values are presented as mean ± SD. FSH, Follicle-stimulating hormone; LH, Luteinizing hormone; SHBG, Sex hormone-binding globin. For estradiol values below the detection level (<15 nmol/L), a value of 15 nmol/L was used when calculating the average group mean. Post values are from the last day of the hormonal treatment period. P-values at 0.05 or below are reported in bold.*

### Strength Measures

#### MVIC, RFD, and EMG

MVIC (knee flexion and extension) increased during the intervention period in both groups, with no significant difference in improvements between groups (Table 4). The results showed no difference in either RFD or EMG after the training intervention or between groups (data not shown).

**TABLE 4:** Strength measures.

	**Estrogen (*n* = 15)**					**Placebo (*n* = 16)**						
	**Pre**		**Post**		***P*−value**	**Pre**		**Post**		***P*-value**	**Training**	**Interaction**
MVIC Ext (NM)	143	± 23.2	161.6	± 30.2	**<0.01**	135.2	± 38.8	158.4	± 39.0	**<0.01**	**<0.01**	0.43
MVIC Flx (NM)	70.0	± 13.8	76.9	± 16.5	**0.03**	66.6	± 20.5	78.8	± 23.2	**<0.01**	**0.03**	0.19
5 RM leg press (kg)	83.8	± 13.6	132.2	± 19.8	**<0.01**	89.1	± 25.4	131.8	± 29.5	**<0.01**	**<0.01**	0.23
5 RM knee Ext (kg)	40.3	± 10.0	57.8	± 11.0	**<0.01**	44.1	± 8.7	59.4	± 11.2	**<0.01**	**<0.01**	0.31
5 RM knee Flx (kg)	18.6	± 3.8	26.9	± 4.8	**<0.01**	20.6	± 4.0	28.4	± 6.4	**<0.01**	**<0.01**	0.26

*Values are presented at mean ± SD. MVIC Ext, maximal voluntary isometric contraction in knee extension; MVIC Flx, maximal voluntary isometric contraction in knee flexion; 5 RM, five-repetition maximum. P-values relates to pre vs. post changes within each group and results from Linear mixed model analysis on training effect and training x treatment interaction effects. P-values at 0.05 or below are reported in bold.*

#### Five RM

Both groups increased their 5 RM strength significantly compared with baseline (34–59%, *p* < 0.001) with no difference in improvement between groups (Table 4).

### Functional Tests

**FIGURE 4:**
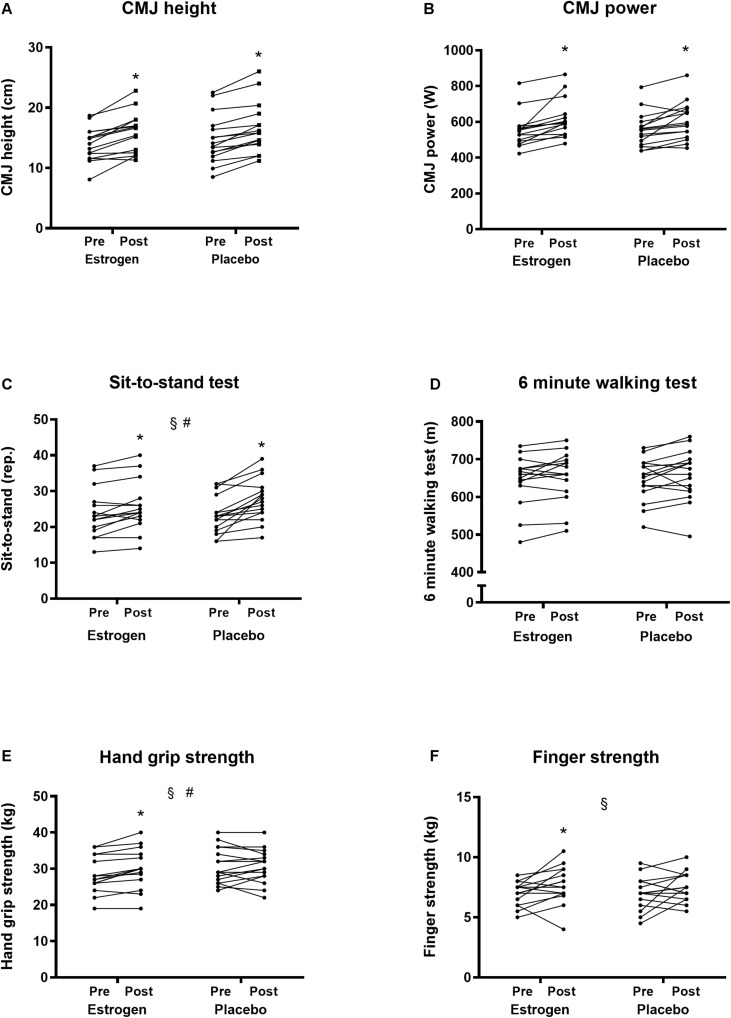
**(A)** Countermovement jump height. **(B)** Power in countermovement jump. **(C)** Sit-to-stand-test. **(D)** Six minutes walking test. **(E)** Handgrip strength. **(F)** Finger strength before and after 12 weeks of resistance training in the individual participants in the ET and PLC group, respectively. ^#^Group × time interaction (*p* < 0.05). ^§^ Training effect (*p* < 0.05). *Different from pre in the individual group (*p* < 0.05).

Results from the functional tests are shown in Figure 4. *Maximal CMJ height* (ET: 13.9 ± 2.8 to 15.9 ± 3.3 cm, PLC: 14.7 ± 4.0 to 16.5 ± 4.1 cm) *and power* (ET: 554.2 ± 96.8 to 617.6 ± 107.3 cm, PLC: 556.1 ± 94.7 to 606.0 ± 104.6 cm) were both significantly improved (∼12–14%) after the training intervention, but no significant differences in improvement were observed between groups (*p* = 0.68 and *p* = 0.44, respectively). *Maximal handgrip strength* was significantly enhanced after the training intervention period in the ET group (28.3 ± 5.1 to 30.0 ± 5.5 kg, *p* < 0.01) but not in the PLC group (30.7 ± 4.9 to 30.7 ± 4.6 kg, *p* = 0.99, training × treatment interaction *p* < 0.05). *Sit-to-stand test* performance was significantly improved after the training intervention (*p* < 0.05). The increase in performance was significantly greater in the PLC group (23.4 ± 5.2 to 27.6 ± 5.9 rep.) (17.9%) compared with the ET group (23.9 ± 6.9 to 25.7 ± 6.9 rep.) (7.5%) (training × treatment interaction *p* < 0.05). *Six minutes walking test* performance was not significantly improved after the training intervention period (*p* = 0.12, ET: 644.2 ± 68.5 to 655.2 ± 67.6 m, PLC: 646.3 ± 56.5 to 658.1 ± 67.0 m.). *Maximal finger strength* was improved after the training intervention (*p* < 0.05), but no training × treatment interaction was observed (*p* = 0.57). Notably, the ET group experienced a significant increase in maximal finger strength (7.0 ± 1.0 to 7.7 ± 1.6 kg, *p* < 0.05), which was not observed for the PLC group (7.0 ± 1.3 to 7.5 ± 1.2 kg) (*p* = 0.14).

**FIGURE 5:**
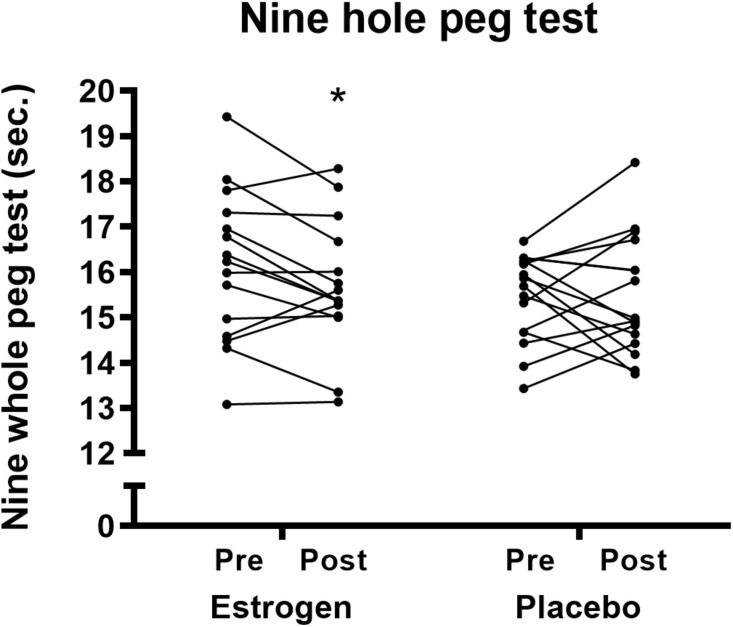
Nine hole peg test before and after 12 weeks of resistance training in the individual participants in the ET and PLC group, respectively. A lower time indicates an improvement. *Different from pre in the individual group (*p* < 0.05).

*Nine-hole-peg-test performance* tended to be improved after the training intervention (*p* = 0.08), but no significant training × treatment interaction was observed (*p* = 0.22), although the ET group experienced a significant improvement compared with baseline (16.1 ± 1.7 to 15.7 ± 1.4 s, *p* < 0.05), whereas this was not experienced by the PLC group (15.5 ± 1 to 15.5 ± 1.3 s) (*p* = 0.98) (Figure 5).

### Food Registration and Physical Activity Level

#### Food Registration

Only 11 subjects (ET *n* = 5, PLC *n* = 6) reported valid food registration data based on their reported daily energy intake (EI/BMR > 1.06) in both registration periods. Data for these 11 persons showed no significant difference (*p* > 0.05) in daily energy intake between the two registration periods. Therefore, data for the two periods were averaged and are presented in Table 5. Daily energy intake, percent of daily energy intake (E%) from carbohydrate, and fat did not differ between groups. There was a significantly greater relative intake of protein (E%) in the PLC group (*p* < 0.05). However, there was no significant difference in protein intake in grams/day and protein in grams/kilogram/BW/day between groups.

**TABLE 5:** Dietary intake.

**Nutrition data**	**Estrogen (*n* = 5)**	**Placebo (*n* = 6)**	***P*-value**
Energy (kJ/day)	7,674.4 ± 1,495.0	7,928.7 ± 1,493.4	0.69
CHO (E%)	47.8 ± 5.6	45.2 ± 5.9	0.29
Fat (E%)	35.4 ± 5.3	35.8 ± 5.9	0.88
Protein (E%)	16.8 ± 2.1	19.0 ± 2.4	**0.03**
Protein (g/day)	61.9 ± 24.5	56.6 ± 18.8	0.55
Protein (g/kg/BW/day)	1.0 ± 0.5	0.9 ± 0.3	0.41

*Values are presented as mean ± SD. BW, Body weight; E%: % of total daily energy intake. P-values represent results from unpaired Student’s t-test (between groups). P-values below 0.05 are reported in bold.*

#### Physical Activity Level

At baseline, there was no significant difference between groups in habitual physical activity level measured by an accelerometer (*p* > 0.05) (data not shown). During the last week of the intervention period, no significant difference in physical activity level was observed compared with the registration before the intervention in either the PLC or the ET group.

## Discussion

To our knowledge, this is the first randomized controlled study to investigate the effect of transdermal estrogen therapy on the response to supervised resistance training in postmenopausal women. The main finding of the present study was that 12 weeks of progressive resistance training combined with transdermal ET led to greater muscle growth than resistance training itself in early postmenopausal women. Both the increase in muscle CSA (quadriceps femoris) measured by MRI and the increase in whole-body FFM measured by DXA were significantly greater in the ET group compared with those in the PLC group. Thereby, our findings suggest that adaptations to an anabolic stimulus such as resistance training are amplified when circulating 17β-estradiol is enhanced by ET.

### Muscle Mass

A recent meta-analysis based on 12 randomized clinical trials, with a total of 4,474 postmenopausal women, concluded that the use of HT without training was not associated with any beneficial or detrimental effect on muscle mass after a median follow-up of 2 years (Javed et al., 2019). However, the risk of bias was reportedly high in 6 of 12 studies and unclear in the further four studies. Data from a cross-sectional study by Ronkainen et al. (2009) support a positive effect of HT on muscle mass (Ronkainen et al., 2009). They included monozygotic twin pairs; one twin was a user of HT, and the other twin was not and reported that the relative muscle area of the thigh was on average 8% larger in the HT users than in the nonusers (Ronkainen et al., 2009). Furthermore, Sørensen et al. (2001) conducted a randomized controlled crossover trial where 16 postmenopausal women (55 ± 3 years) went through two periods of 12 weeks with either HT or placebo (Sørensen et al., 2001). During the placebo period, the women lost lean body mass (LBM) (−1.0 kg), whereas LBM was maintained in the HT period (+ 0.3 kg). The latter observations indicate that HT without a concurrent training intervention may at best reduce muscle loss. However, in the present resistance training study, we aimed at increasing muscle mass and elucidate how ET influences the response to resistance training. Studies investigating the effect of resistance training combined with HT in postmenopausal women are few (Heikkinen et al., 1997; Sipila et al., 2001). In a 1 year randomized controlled trial, Sipila et al. (2001) studied the effect of HT and high impact exercise training alone and combined on body composition and physical function in postmenopausal women. Lean tissue CSA (quadriceps femoris) in the non-training groups was significantly higher in the HT group (6.3%) compared with the placebo group (0.7%) after the 1 year intervention. In the same study, training + HT increased muscle CSA in quadriceps femoris and the lower leg by 7.1 and 9.1%, respectively, compared with 2.2 and 3.0%, respectively, in the training-only group. The latter results suggest a synergistic effect of HT and training on gains in lean muscle mass and is in line with our findings of a superior effect on muscle growth in response to training in the ET group. However, Sipila et al. (2001) did only observe a tendency toward a greater increase in lean tissue CSA (quadriceps) in the HT + training group compared with the HT-only group after 6 months of intervention (*p* = 0.055), which was not present after 12 months (*p* = 0.63) (Sipila et al., 2001). Likewise, the increase in whole-body LBM after 12 months in the HT + training group was not significantly different from the HT-only group, indicating that training does not add to the effect of HT. The discrepancy to our data might be explained by their training program, which was not designed to induce muscle hypertrophy, and the majority of sessions were performed at home, which makes it difficult to control for compliance during a whole year. This is underlined by the increase in lean CSA, which was only 2–3% after 12 months of training in the training-only group compared with 4–5% in the PLC group after 12 weeks of supervised resistance training in the present study. A similar limitation is coupled to the trail by Heikkinen et al. (1997), who performed a 2 years prospective study where postmenopausal women were randomized to receive HT or placebo. Furthermore, half of each group were advised to exercise two times at home and participate in one supervised training session each week. The training was designed to improve bone mass density and focused on loading the lumbar and femoral areas. HT significantly improved flexion strength in the upper body (∼2–5 kg), whereas exercise only marginally improved flexion strength (∼2 kg) during the 2 years period compared with placebo. No interaction effect was reported, and muscle mass data were not provided (Heikkinen et al., 1997). Therefore, our present study is the first, which has specifically aimed to elucidate the effect of ET on muscle growth when combined with a resistance training program designed to induce muscle hypertrophy.

An increase in muscle mass is induced when the average myofibrillar protein synthesis rate is higher than myofibrillar protein breakdown over a while. A previous study by Hansen et al. (2012) suggests a positive effect of combining ET and resistance exercise on myofibrillar protein synthesis fractional synthesis rate (FSR). Twenty postmenopausal women, 10 hysterectomized using ET and 10 controls, all performed 10 sets of 10 repetitions of one-legged knee extension at 10 RM. Twenty-four hours later, muscle FSR was measured in both the exercised and resting leg. In the ET group, myofibrillar protein FSR was significantly higher in the exercise leg than in the non-exercised leg. In the controls, no difference in myofibrillar protein FSR between legs was observed, thus indicating that ET enhances the sensitivity to anabolic stimuli by enhancing the myofibrillar protein FSR and thereby inducing a positive muscle protein balance. Moreover, estrogen has been shown to positively affect the muscle tissue stem cells, the satellite cells (Kitajima and Ono, 2016). During hypertrophy, satellite cells play an important role in regeneration and repair and are the donors in regard to increasing the number of myonuclei (Dumont et al., 2015). In a study conducted in ovariectomized mice (OVX), Kitajima and Ono (2016) observed that force and muscle mass was reduced after ovariectomy. When treating half of the mice with estrogen, a regain of force, and muscle mass was observed in the OVX + ET mice, which was not observed in the OVX mice. The authors attributed their findings to an observed reduction in activated satellite cells in the OVX mice, which probably hampered the regain of muscle strength and mass. However, more studies are needed to elucidate the specific molecular mechanisms behind the enhanced gain in muscle mass during training with estrogen supplementation. At the same time, it is important to emphasize that both groups experienced an increase in muscle mass after the training period, indicating that postmenopausal women can increase their muscle mass by performing regular resistance training both with and without ET. Resistance training leads to a reduction in the MHC IIx (Andersen and Aagaard, 2000). Our data showed significant downregulation of MHC IIx protein in the PLC group and a numeric downregulation in the ET group compared with baseline. This underlines that the training regime effectively induced training specific molecular adaptations within the muscle regardless of treatment with ET. However, at baseline, we observed different content of MHC I, with a higher percentage in the PLC group (PLC = 58%, ET = 48%). A study by Häkkinen et al. (1998) showed, in a small sample size (*n* = 14), a correlation (*r* = −0.56) between the percent of MHC I at baseline and the change in CSA in percent after 10 weeks of supervised resistance training three times per week in young and old men. Therefore, we cannot exclude the possibility that the MHC content has played a role in the result. On the other hand, the muscle biopsy only reflects a very small area of vastus lateralis, why we cannot assure that the same distribution is seen in quadriceps femoris overall.

### Muscle Strength, Muscle Function, and Physical Performance

Muscle strength, muscle function, and physical performance tests were included as secondary outcome parameters in the present study. Overall, improvements in performance parameters were observed after the 12 weeks resistance-training period compared with baseline in MVIC (both leg Ext and Flx), five RM tests, CMJ, handgrip strength, sit-to-stand test, and nine-hole peg test but not 6 min walking test (*p* = 0.08). We observed a significantly greater improvement in the ET group compared with PLC in the handgrip test (interaction *p* = 0.02). Furthermore, an improvement in the finger strength test was only observed in the ET group (*p* = 0.02) but not in PLC (*p* = 0.14). The improvement of hand and finger strength in the ET group is interesting because our training program was not designed to particularly improve hand and finger strength parameters. The observed improvement in the ET group may be related to a direct effect of ET independent of training. In line with our observations, positive effects of HT on muscle strength without a concurrent training intervention have previously been reported (Phillips et al., 1993; Heikkinen et al., 1997; Skelton et al., 1999). Skelton et al. (1999), in a 6–12 months randomized controlled trial with 102 postmenopausal women, observed a significant increase in maximal abductor pollicis muscle strength in the HT group, whereas the placebo group experienced a decline in muscle strength during the intervention period. The participants in the studies mentioned earlier received a combination of estrogen and progesterone. Although we observed significant increases in muscle strength and when only administrating estrogen, it can be suggested that it is estrogen that induces the beneficial effect on muscle strength. A limitation to the present study design was the lack of non-training groups with and without ET. This would have allowed us to investigate whether the positive effect of ET + training on muscle growth was superior compared with the effect of ET alone.

Despite differences in muscle mass gain between groups, we did not observe any superior effect of ET on MVIC, five RM tests, CMJ, sit-to-stand test, and 6 min walking test. Generally, differences in functional parameters between groups can be difficult to detect for several reasons. Firstly, our study participants were generally healthy and therefore performed very well in the functional tests (sit-to-stand and 6 min walking test) primarily intended for the test of elderly and more frail individuals (Alcazar et al., 2018). Secondly, the effect of training interventions on MVIC, five RM, and CMJ in previously untrained participants is substantial, making it difficult to detect a hormonal treatment effect on top of this improvement. Thirdly, the sensitivity for detecting a small difference is low in relative complex tests such as CMJ and the nine-hole peg test, why a second familiarization session would be have been beneficial.

The greater improvement in the sit-to-stand test in the PLC group seems contradictory to the greater improvement in muscle mass in the ET group. There may be multiple reasons for the differences in the sit-to-stand improvement. The sit-to-stand test is a technical and relatively complex test compared with the test of isometric maximal strength. Therefore, improvements in performance in the sit-to-stand test may for persons not sufficiently habituated to the test relate to improvements in technical skills rather than relate to improvement in lower limb strength. In support, the improvements in the sit-to-stand test were not associated with changes in isometric strength. In addition, the risk of random changes in a more technically difficult test is higher, especially when the sample size, as in the present trial, is relatively small. We only included one familiarization test, but potentially a second familiarization test would have limited the variations in improvements between groups.

The nine-hole peg test is used to measure finger dexterity in patients with various neurological diagnoses, but a general reduction in performance in the test is also observed during aging (Mathiowetz et al., 1985). Interestingly, we found a significant improvement in the nine-hole peg test only in the ET group. This could be in line with our findings in handgrip and finger strength, which all together could be an indication of an improved neuromuscular function due to the ET treatment.

### Methodological Considerations

The present study had several strengths in regard to the design and methods. (1) All training sessions were supervised one-to-one, and the compliance to the training program was 100%. (2) All participants in our study had their last bleeding period within the last 5 years. Of those, four women had their last bleeding period between 6 and 12 months before the intervention start and were therefore officially not fulfiling the definition of menopause being at least 1 year since the last bleeding period. However, all participants fulfiled the criteria of an FSH level of > 30 (IU/L), and the inclusion of early postmenopausal women, was in line with the recommendations for hormonal treatment from the International Menopause Society (Baber et al., 2016). Several studies have included women from early menopause to women in their eighties, which complicate the conclusions that can be drawn from the study, as a difference in response to HT between early and late menopausal women on muscle protein breakdown markers has been reported (Park et al., 2019). Furthermore, others reported a lower concentration of estrogen receptors in skeletal muscle in late menopausal women, which may explain the difference in response to ET/HT treatment (Park et al., 2017). (3) None of the participants was smokers or had any history of cancer. These health parameters are important to take into consideration when applying estrogen treatment in this population. (4) The women in the ET group received the same type and dose of ET, allowing us to study the isolated effect of estrogen replacement rather than a combination of estrogen and gestagen, such as in HT.

Unfortunately, there is also a critical point related to the present data set. The protein intake in the present study was self-reported, and only one-third of the participants reported a plausible valid energy intake in accordance with an energy-balanced diet (Goldberg et al., 1991). It is a known phenomenon that participants in research studies unconsciously or consciously underreport their energy intake (Bokhof et al., 2010). From the 11 participants, we obtained valid data from an intake of 1.0 (ET) and 0.9 (PLC) gram of protein/kilogram body weight (BW)/day was reported with no difference between groups. The reported protein intake is acceptable compared with the recommendations for the general population (0.83 g protein/kg/BW/day) (Carbone and Pasiakos, 2019). However, the reported protein intake is in the low end, when compared with recommended daily protein intake for supporting muscle growth during resistance training in men (1.6 g protein/kg BW/day, 95% c.i. 1.2–2.2 g protein/kg/BW/day) (Morton et al., 2018). The optimal intake of protein/kilogram/day for optimizing muscle hypertrophy may be lower in women due to a relative lower muscle mass/kilogram BW. Still, we cannot exclude that a higher daily protein intake may have been optimal to support muscle growth (Morton et al., 2018). Moreover, the dietary recording was only done twice for 4 days at a time. The dietary recording is a time-consuming process, and even with few days of registering, we experienced a great deficiency in the data due to non-realistic reporting of consumption. As already mentioned, data are only included from 11 subjects, as the rest reported data, which was not plausible in reference to Goldberg’s cutoff limit (Goldberg et al., 1991; Bokhof et al., 2010).

Rizzoli et al. (2014) underline the importance of sufficient intake of vitamin D (800 IU/day) to obtain a level of > 50 nmol/L and calcium (1,000 mg/day) to prevent age-related deterioration of skeletal muscle. We have no reliable data for the participants’ vitamin D and calcium intake. However, at baseline, we measured all participants’ vitamin D status (25-hydroxy vitamin D) and circulating calcium concentration, and only women who had values within the normal range (hospital reference values) were included as participants. No different was seen between groups for either 25-hydroxy vitamin D (ET: 83.2 ± 13.9, PLC: 88.7 ± 24.4 nmol/L) (*p* = 0.43) or calcium (ET: 2.3 ± 0.1, PLC: 2.4 ± 0.1 mmol/L). Future studies should pay attention to the attained levels of sex hormones in the ET group. As expected, the estradiol level was unchanged in the PLC group, whereas it rose significantly in the ET group (318 pmol/L). In comparison, the estradiol level during a normal menstrual cycle in premenopausal women fluctuates between 240 to 2,400 pmol/L. Although the participants in the ET group received a rather substantial dose of estradiol, FSH and LH levels did not reach premenopausal levels (< 15 IU/L), and thus it can be speculated that a higher dose of estradiol would have led to even more pronounced effects on muscle mass and function. Future studies should focus on individual estrogen treatment regimens making sure that all participants obtain the necessary dose to reach a comparable level between participants. In combination with enhancing the sample size, this would probably have contributed to more pronounced effects.

## Conclusion

The present study demonstrates that a 12 weeks resistance-training period induced a greater gain in muscle mass and handgrip strength in early postmenopausal women treated with transdermal ET compared with PLC. Future training studies should compare the isolated effects of transdermal ET, training and the combined intervention, respectively, to clarify if the combination of training and ET induce an amplified positive effect on skeletal muscle mass and strength compared to training or ET alone.

## Data Availability Statement

The raw data supporting the conclusions of this article will be made available by the authors, without undue reservation.

## Ethics Statement

The studies involving human participants were reviewed and approved by the Committee of Region Midtjylland. The participants provided their written informed consent to participate in this study.

## Author Contributions

TD, CG, and MH conceived and designed the study. TD, FJ, MB, MM, KL, and MH carried out the experiments. TD, LD, NØ, SR, and MH analyzed the samples and data. TD, LD, SR, CG, and MH interpreted the results of the experiments. TD, LD, and MH prepared the figures and drafted the manuscript. All authors edited, revised, and approved the final version of the manuscript.

## Conflict of Interest

The authors declare that the research was conducted in the absence of any commercial or financial relationships that could be construed as a potential conflict of interest.
